# Comparison of bioresorbable vs durable polymer drug-eluting stents in unprotected left main (from the RAIN-CARDIOGROUP VII Study)

**DOI:** 10.1186/s12872-020-01420-5

**Published:** 2020-05-15

**Authors:** Mario Iannaccone, Umberto Barbero, Michele De Benedictis, Yoichi Imori, Giorgio Quadri, Daniela Trabattoni, Nicola Ryan, Giuseppe Venuti, Andrea Montabone, Wojciech Wojakowski, Andrea Rognoni, Gerard Helft, Radoslaw Parma, Leonardo De Luca, Michele Autelli, Giacomo Boccuzzi, Alessio Mattesini, Christian Templin, Enrico Cerrato, Wojciech Wańha, Grzegorz Smolka, Zenon Huczek, Francesco Tomassini, Bernardo Cortese, Davide Capodanno, Alaide Chieffo, Ivan Nuñez-Gil, Sebastiano Gili, Antonia Bassignana, Carlo di Mario, Baldassarre Doronzo, Pierluigi Omedè, Maurizio D’Amico, Delio Tedeschi, Ferdinando Varbella, Thomas Luscher, Imad Sheiban, Javier Escaned, Mauro Rinaldi, Fabrizio D’Ascenzo

**Affiliations:** 1 Division of Cardiology, SS. Annunziata Hospital, ASL CN1, Savigliano, Italy; 2 Department of Cardiovascular Medicine, Nippon Medical School, 1-1-5, Sendagi, Bunkyo-ku, Tokyo, Japan; 3 Department of Cardiology, Infermi Hospital, Rivoli, Italy; 4 Department of Cardiology, San Luigi Gonzaga Hospital, Orbassano, Turin, Italy; 5 Department of Cardiovascular Sciences, IRCCS Centro Cardiologico Monzino, Milan, Italy; 6 University of Milan, Milan, Italy; 7 Department of Cardiology, Hospital Clinico San Carlos, Madrid, Spain; 8 Division of Cardiology, Cardio-Thoracic-Vascular Department, Azienda Ospedaliero Universitaria “Policlinico-Vittorio Emanuele,”, Catania, Italy; 9 Structural Interventional Cardiology, Careggi University Hospital, Florence, Italy; 10 Department of Cardiology, Medical University of Silesia, Katowice, Poland; 11 Coronary Care Unit and Catheterization Laboratory, A.O.U. Maggiore della Carità, Novara, Italy; 12 Division of Cardiology, Pierre and Marie Curie University, Paris, France; 13 University Clinical Hospital, Warsaw, Poland; 14 Pederzoli Hospital, Peschiera del Garda, Italy; 15 Cardiology Department, Ospedale San Giovanni Bosco, Turin, Italy; 16 Division of Cardiology, Universityspirtal of Zurich, Zürich, Switzerland; 17 Interventional Cardiology, ASST Fatebenefratelli-Sacco, Milan, Italy; 18 San Raffaele Scientific Institute, Milan, Italy; 19 Division of Cardiology, Department of Internal Medicine, Città della Salute e della Scienza, Turin, Italy; 20 Interventional Cardiology, Istituto clinico Sant’anna, Brescia, Italy; 21 Interventional Cardiology, Pederzoli Hospital Peschiera del Garda, Verona, Italy

**Keywords:** Percutaneous coronary intervention, Drug eluting stents, Struts thickness, Left main, Coronary bifurcation

## Abstract

**Background:**

There are limited data regarding the impact of bioresorbable polymer drug eluting stent (BP-DES) compared to durable polymer drug eluting stent (DP-DES) in patients treated with percutaneous coronary intervention using ultrathin stents in left main or bifurcations.

**Methods:**

In the RAIN registry (ClinicalTrials NCT03544294, june 2018 retrospectively registered) patients with a ULM or bifurcation stenosis treated with PCI using ultrathin stents (struts thinner than 81 μm) were enrolled. The primary endpoint was the rate of target lesion revascularization (TLR); major adverse cardiovascular events (MACE, a composite of all-cause death, myocardial infarction, TLR and stent thrombosis) and its components, along with target vessel revascularization (TVR) were the secondary ones. A propensity score with matching analysis to compare patients treated with BP-DES versus DP-DES was also assessed.

**Results:**

From 3001 enrolled patients, after propensity score analysis 1400 patients (700 for each group) were selected. Among them, 352 had ULM disease and 1048 had non-LM bifurcations. At 16 months (12–22), rates of TLR (3.7% vs 2.9%, *p* = 0.22) and MACE were similar (12.3% vs. 11.6%, *p* = 0.74) as well as for the other endpoints. Sensitivity analysis of outcomes after a two-stents strategy, showed better outcome in term of MACE (20.4% vs 10%, *p* = 0.03) and TVR (12% vs 4.6%, *p* = 0.05) and a trend towards lower TLR in patients treated with BP-DES.

**Conclusion:**

In patients with bifurcations or ULM treated with ultrathin stents BP-DES seems to perform similarly to DP-DES: the trends toward improved clinical outcomes in patients treated with the BP-DES might potentially be of value for speculating the stent choice in selected high-risk subgroups of patients at increased risk of ischemic events.

**Trial registration:**

ClinicalTrials.gov Identifier: NCT03544294. Retrospectively registered June 1, 2018.

## Background

The treatment of unprotected left main and of coronary bifurcation still represents a challenge for interventional cardiologists due to both procedural complications and higher restenosis rates compared with non-bifurcation lesions [1–4].

The complexity of the bifurcation *milieu* is rooted into the unique flow patterns that characterized them, with local low and oscillatory endothelial shear stress along the lateral walls of the main vessel and of the side branch, whereas high endothelial shear stress develops at the carina. This ultimatey leads to a prothrombotic and atherogenic flow-pattern, and even after treatment with percutaneous coronary intervention (PCI), it may increase the failure rate wit need for subsequent revascularization on the target lesion (TLR) and an increased risk of stent thrombosis (ST) [4–6].

In the last years, BP-DES (Bioresorbable polymer drug eluting stents) have been introduced with the rationale to potentially decrease ST. Differently from durable polymer stents (DP), after the elution of the antiproliferative drug the bioreabsorbable polymer is going to dissolve leaving behind a bare metal stent, thus reducing the local inflammatory reactions and therefore the risk of thrombosis related to a permanent polymer. Clinically, this translated into lower rates of TLR for BP-DESs implanted in coronary bifurcations in the LEADER-FREE [7], although this RCT was weakened by the comparison with a first generation stent. Regarding currently implanted second generation DES, both in the EVOLVE II trial and in a recent paper of Mennuni et al. [8, 9], BP-DESs were shown to be safe and effective as compared to durable polymer, although coronary bifurcations and LM were underrepresented (respectively about 4 and 15%) [10].

In light of the intrinsic limitations of the above studies, the RAIN study (very thin stents for patients with MAIN or bifurcation in real life: the RAIN, a multicenter study) was designed to evaluate the clinical performance of ultrathin stents in everyday clinical practice. We here present an analysis of the RAIN study aimed to evaluate the safety and efficacy of BP-DES in the bifurcation setting.

## Methods

The RAIN is a large multicenter retrospective observational registry (ClinicalTrials NCT03544294, retrospectively registered; see Additional file 1 for enrolling sites). The study was conducted in accordance with the ethical principles of the Declaration of Helsinki and are consistent with ICH Good Clinical Practice as well as regulatory requirements. It was approved by an institutional review committee, and all patients provided informed consent.

### Inclusion criteria

The RAIN registry included all consecutive patients from June 2015 to January 2017 undergoing complex PCI involving LM and/or bifurcation with ultrathin stents (Promus Element, Xience Alpine, Ultimaster, Synergy, and Resolute Onyx; see Additional file 1 for more details on the stents involved in this study).

### Baseline and procedural data

Cardiovascular risk factors, clinical presentation, angiographic features, use of IntraVascular UltraSound (IVUS), Optical Coherence Tomography (OCT) and Fractional Flow Reserve (FFR) were recorded, along with the characteristics of the implanted stents. IVUS or OCT was used prior to stent implantation to assess the severity of the stenosis and side branch involvement, and post stent implantation to evaluate dissection and the requirement for stent optimisation. The decision to post-dilate, to perform final kissing balloon (FKB), to assess intracoronary imaging and the choice of the stenting technique (provisional versus 2-stent), was at the discretion of the treating physician.

The above data were derived from electronic patient records at each center, while follow-up data were obtained from clinical assessment, telephonic consultations or via primary care physicians and then recorded online (http://www.cardiogroup.org/RAIN/index.php?cat=home).

### Endpoints

The rate of TLR was the primary endpoint, while MACE (a composite endpoint of all-cause death, myocardial infarction, TLR and stent thrombosis) and its components, along with TVR were the secondary endpoints. The analyses were performed according to PCI strategy (provisional vs two-stent).

### Statistical analysis

Categorical variables are reported as count and percentages, whereas continuous variables as mean and standard deviations or interquartile range (IQR). Gaussian or not Gaussian distribution was evaluated by Kolmogorov-Smirnoff test. The t-test has been used to assess differences between parametric continuous variables, Mann-Whitney U test for non parametric variables, the *chi*-square test for categorical variables and Fisher’s exact test for 2 × 2 tables. The a priori statistical significance level was set at α = 0.05. To account for clustered data among centres we used a normal regression (ANOVA) approach with a fixed effect for cluster and an effect for group when data were normally distributed. For non-normal data, we used the Wilcoxon rank sum test modified to account for clustering. For propensity score, first logistic regression analysis was done for all baseline features that differed between BP-DES and DP-DES, matching was computed after division into quintiles and methods of the 1:1 nearest neighbor on the estimated propensity score [11]. Calibration was tested with Hosmer-Lermeshow, and accuracy was assessed with Area Under the Curve. Standardized differences were evaluated before and after matching to evaluate the performance of the model. All statistical analyses were performed with SPSS 21 and differences were considered significant at α = 0.05.

## Results

### Before propensity score with matching

**Fig. 1:**
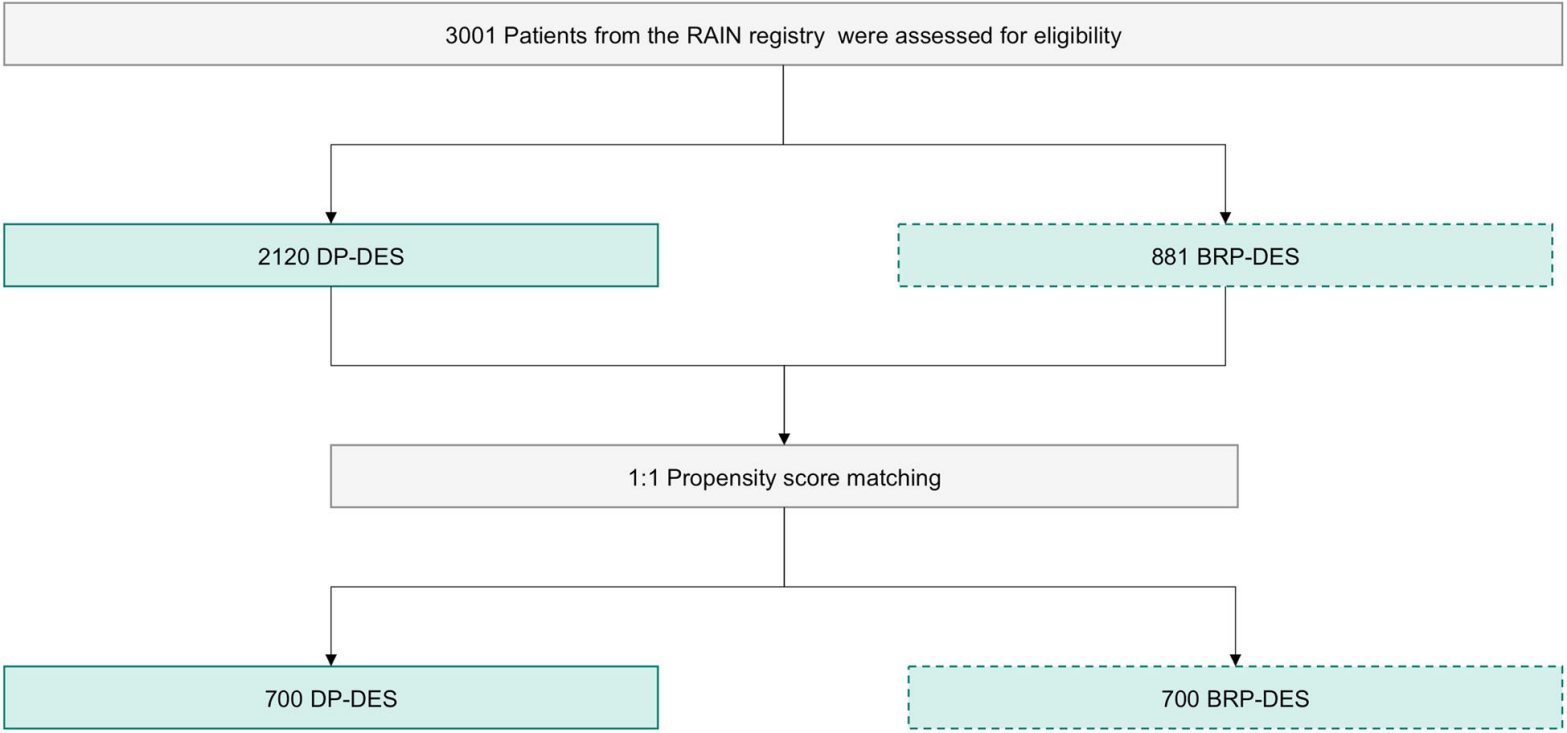
design of the study

At the end of the enrolling period 3001 patients had been recorded: 2120 were treated with DP-DES ad 881 with BP-DES (see Fig. 1).

At baseline patients in the BP-DES were more hypertensive (28.8% vs 23.2% in the DP group, *p* < 0.01), with a higher rate of previous MI (35.3% vs 26.6% in the DP group, *p* < 0.01) and they were more often admitted due to STEMI (19.1% vs 16%, *p* < 0.01). On the other side, patients with DP-DES were more often hyperlipidemic (58.1% vs. 54.6% in the BP group, *p* < 0.01; see Additional file 1: Table A).

Patients with BP-DES more frequently presented diffuse coronary disease (55.1% vs 29.9%% in the DP group, *p* < 0.01), and with true bifurcation involvement (i.e. Medina 1,1,1 or 0,1,1; 28.1% in the BP group vs 17.5% in the DP group, *p* < 0.001). They were less frequently treated with two-stent technique (76.9% vs 82.4% in the DP group, *p* < 0.01). Finally, there was a similar rate of total ULM disease (30% vs 26.9%, *p* = 0.45) (see Additional file 1: Table B).

### After propensity score with matching

After multivariate adjustment, 700 patients for each group were selected. Baseline clinical features were comparable, with similar rates of presentation for STEMI (19% in the DP group vs. 18.1% in the BP group, *p* = 0.1, see Table 1 and Fig. 1).

**Table 1:** Baseline Characteristics Post PSWM

	DP-DES (700 pt.)	BP-DES (700 pt.)	***P***
*Age (mean* ± SD)	70.7 ± 9	70.7 ± 10	0.97
*Female (%)*	20.5	23.1	0.24
*Hypertension (%)*	77.1	73.8	0.17
*Hyperlipidemia (%)*	62	63.8	0.5
*Diabete mellitus non ID (%)*	26.2	27.8	0.5
*Diabete mellitus ID (%)*	6.4	9.5	0.09
*Previous smoker (%)*	31.8	30.3	0.5
*Renal Disease (gfr < 60 ml/min/m2) (%)*	19.7	19.9	0.71
*Previous PCI (%)*	32	33.6	0.57
*Previous CABG (%)*	4.9	4.4	0.7
*Previous MI (%)*	31.8	36	0.1
*ASA + Clopidogrel (%)*	64.3	66.4	0.25
*ASA + Ticagrelor (%)*	24.2	23.8	0.22
*ASA + Prasugrel (%)*	7.6	8.0	0.11
*Length of DAPT (months)*	11.3	11.7	0.34
*Indication for PCI: (%)*			0.1
- *STEMI*	*19*	18.1	
- *NSTEMI*	25.4	27.8	
- *UA*	18.2	14.7	
- *Stable angina*	14.9	21.6	
- *Planned angiographic follow up*	7.1	4.9	

At angiography, similar percentages of patients had a LM disease (23.1% in the DP group vs 27.1% in the BP group, *p* = 0.87) and true bifurcation involvement (19.7% in the DP group vs 21.9% in the BP group, *p* = 0.5). Provisional strategy was successfully performed in both groups in the majority of patients (82.1% in the DP group vs. 82.2% in the BP group, *p* = 0.68, see Table 2).

**Table 2:** Interventional Characteristics post PSWM

	DP-DES (700 pt)	BP-DES (700 pt)	***P***
*Radial access (%)*	69.7	68.9	0.77
*Overall LM (%)*	23.1	27.1	0.87
*Site of lesion:*			0.11
- *Ostial LM*	23.9	25.5	
- *Mid LM*	46.8	49.3	
- *Distal LM*	19.9	16.3	
*Type C lesion (%)*	44.3	41.9	0.37
*Severe calcification (%)*	13.2	14	0.68
*Diffuse disease (%)*	52.5	55.9	0.23
*Bifurcation site (%)*			0.13
- *Distal LM*	27.4	30.7	
- *LAD/Dg*	45.9	48.4	
- *LCx/Om*	19.1	15.4	
- *RCA/Pl*	7.5	5.6	
*True bifurcation (medina 1,1,1 or 0,1,1)*	19.7	21.9	0.5
*Provisional strategy (%)*	82.1	82.2	0.9
*2 stents technique strategy (%)*			0.1
- *Culotte*	1.7	1.6	
- *Mini crush*	4.1	5	
- *Crush*	0.5	0.8	
- *DK-crush*	0.3	0.6	
- *T stent*	4.4	2.8	
- *TAP stent*	3.9	3.7	
*Use of imaging:*			0.09
- *IVUS*	29.9	34.2	
- *OCT*	0.9	1.6	

**Fig. 2:**
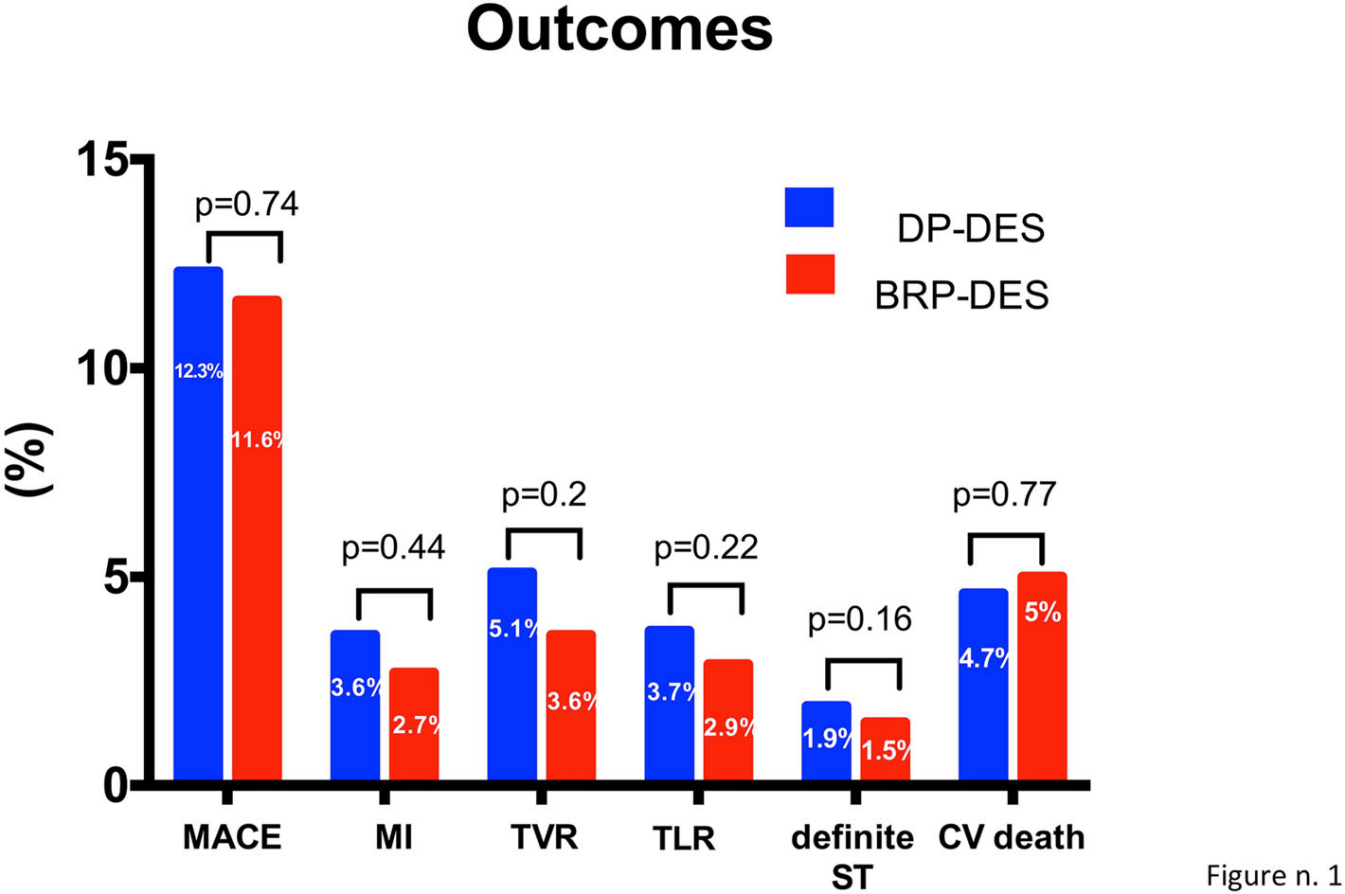
outcomes at follow-up. Blue columns represent the percentage of events among patients receiving a DP-DES, red columns represent the percentage of events among patients receiving a BP-DES

At a median follow up of 16 (12–22) months, rates of TLR (3.7% vs 2.9%, *p* = 0.22) and MACE were similar (12.3% vs. 11.6%, *p* = 0.74), without significant differences among all the secondary endpoint (see Fig. 2).

**Fig. 3:**
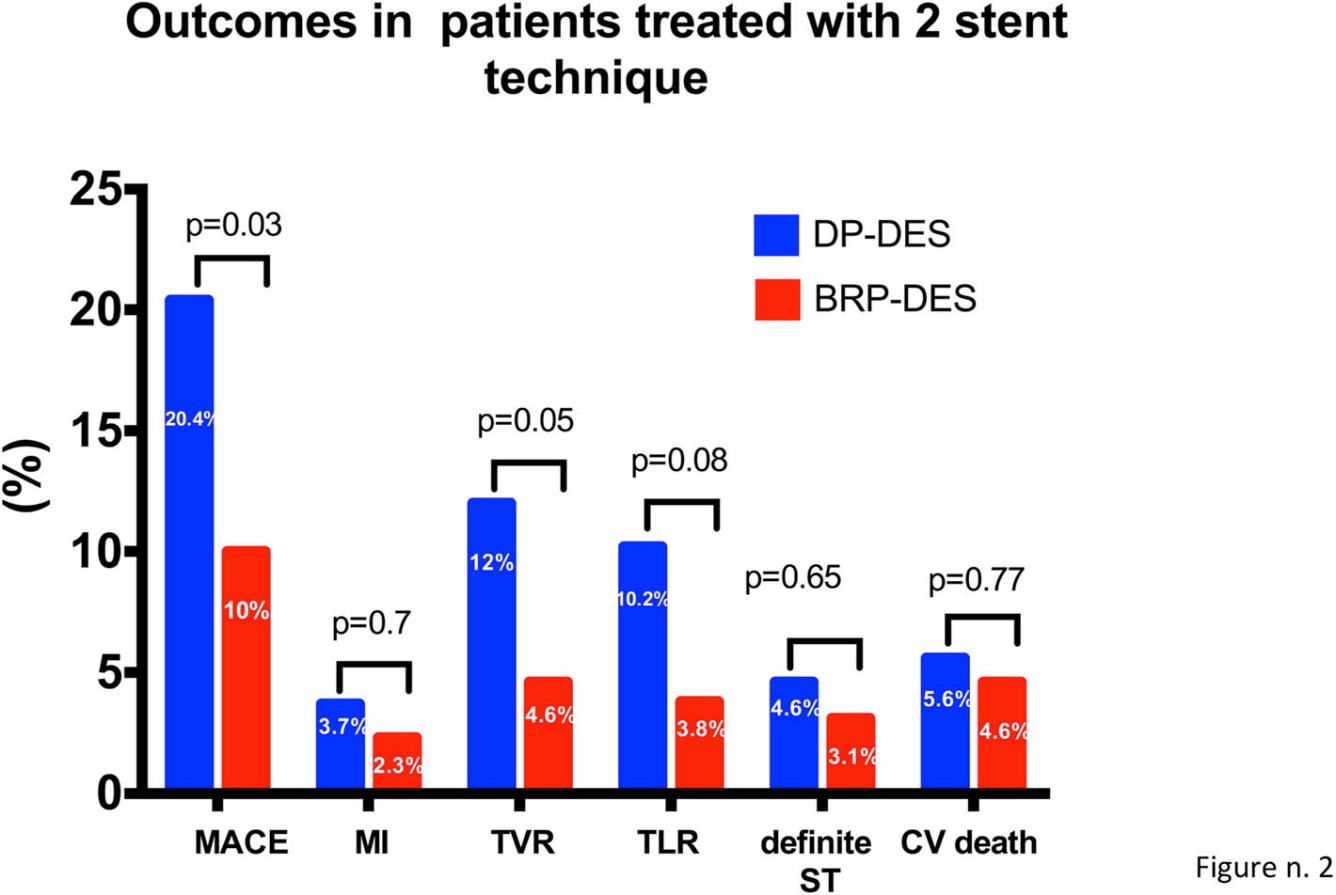
outcomes at follow-up in the subgroup of patients in whom a coronary bifurcation was treated with a 2-stent technique. Blue columns represent the percentage of events among patients receiving a DP-DES, red columns represent the percentage of events among patients receiving a BP-DES

At sensitivity analysis for patients treated with two stents strategy, patients treated with BP-DES showed a better outcome in term of MACE (20.4% vs 10% in the DP group, *p* = 0.03) and TVR (12% vs 4.6% in the DP group, *p* = 0.05, see Fig. 3) and a trend towards TLR (3.7% vs 2.9%, *p* = 0.22).

## Discussion

Our main findings may be summarised as follows:

- Among patients treated for Left Main disease, the risk of MACE was similar for BP-DES and DP-DES.
- Among patients treated with two stents strategy in both LM and non-LM bifurcation involvement, patients treated with BP-DES showed a better outcome in term of MACE and TVR.
- No differences in ST are evident in BP-DES compared to DP-DES group.

To the best of our knowledge, this is the first real-world, observational registry evaluating the safety and efficacy profile of different ultrathin stents (struts thinner than 81 μm) in patients with a ULM stenosis treated with PCI using newer-generation abluminal BP- DES as compared to the DP-DES.

BP-DES have been developed to combine the best of both family of metallic stents, i.e. the efficacy of DES and the late safety associated with BMS. However, the available evidences on cardiac death, MI, or stent thrombosis are still scarce [12–17]: network meta-analyses have indicated an excess risk of BP-DES with regard to MI or stent thrombosis when compared with DP-EES (i.e. Xience, Abbott Vascular, Santa Clara, California), though their results were restricted due to heterogeneity of devices in the BP-DES group and to limited follow-up duration [12–14]. Of note, the meta-analyses by Kang et al. [12] and by Navarese et al. [14] included BP-BES trials using the Biosensors BioMatrix device (Biosensors International, Singapore), the meta-analysis of Bangalore et al. [13] included also trials using an other sirolimus-eluting stent (SES) with biodegradable polymer (Yukon Choice PC, Translumina, Hechingen, Germany) while the one by Cassese et al. [15] included trials on the ultrathin sirolimus-eluting Orsiro device (Biotronik, Bülach, Switzerland).

The principal finding of our current analysis is that BP-DES actually showed a similar safety and efficacy profile at 5 years compared with the DP-DES. Notably - apart from ST - event rates in our study were low keeping into account the clinical and anatomical complexity of the enrolled patients, and similar to previous studies on new-generation DES [16, 17]. These data reflect the global improvement in quality and safety outcomes of these devices mainly due to either the effect of anti-proliferative drug on restenosis and either to the thin struts technology that facilitate the endothelial coverage. However in our study the whole population analysis did not show differences between patients who received BP-DES compared to DP-DES: this might be due to the overwhelming benefit of the thin strut design over the polymer material: the difference in adverse events is in fact observed during period of time that is shorter than the complete polymers dissolution time. These findings highlight the importance of the overall DES design and biocompatibility on the clinical performance of contemporary DES [3]. The effect of stent’s struts thickness has been well established already, with thinner struts showing to produce less inflammation, vessel injury, neointimal proliferation, as well as thrombus formation when compared with thicker ones [18, 19]. Furthermore, the 5-year analysis of the COMPARE II trial also confirmed the early- and mid-term similar safety and efficacy of the BP-BES and the DP-EES, thus challenging the concept itself of the biodegradable polymer coating [20, 21]. On the other hand, Bayesian analysis in the BIOFLOW-V trial, despite limited by the analysis itself and by the particular thickness of the stent used, is encouraging for an actual role of bioresorbable polymers [22, 23]. Therefore, whether BP-DES are as safe and effective as DP-DES should be proven for each specific stent by an appropriately designed clinical trial.

The second interesting result is the trend towards better outcome in term of MACE and TVR in patients treated with BP-DES and a two stents strategy. The higher risk of subsequent events in this challenging anatomical subset is well known, and it is related either to patient either to technique drawbacks [23, 24]. Despite the low percentage of patients included in this analysis (about 20% of the patients selected by propensity score), we can speculate that in an high risk setting like a double stent bifurcation treatment in left main disease, the added value of a bioresorbable polymer in terms of vessel recovery as above explained might really make the difference in terms of outcomes. Of course, this thesis should come on top of more strong assumption about the outcomes related to the use of two stents in bifurcation, most important the technique used [2, 23].

As regard the percentage of stent thrombosis we found, we should not forget that such high numbers are actually in line with other real life reports with comparable follow-up [24]. Furthermore, our study enrolled consecutive all-comers patients, reflecting real practice outcomes that are obviously different from the safer environment of randomized trial. The percentages in the whole population analysed are actually similar to what reported in the COMPARE I trial (A Trial of Everolimus-Eluting Stents and Paclitaxel Stents for Coronary Revascularization in Daily Practice) of 1.8% [25] and of the LEADERS trial (Limus Eluted From a Durable Versus Erodable Stent Coating) of 2.6% [8]. These findings stress the importance of correct identification of critical bifurcation disease by means of fraction flow reserve or intravascular imaging [26, 27] and the value of longer double anti-platelet therapy in patients with higher thrombotic risk [28], especially when this anatomical feature comes together with higher clinical risk situation like ACS [29–31].

### Limitations

There are several limitations to this study. First, this was not a randomized controlled study: with the use of propensity score we could not adjust for variables which were not possible to collect (e.g. the experience of the physicians performing PCI) or others among multiple factors that might be implicated in DES acute thrombogenicity and long-term vascular healing (e.g. the the polymer biocompatibility, its composition and distribution, and -in case of bioresorbable polymers- the duration of bioresorption [3, 17, 21]). It is difficult to assess the different contribution of all these confounding factors in a single study. Furthermore, the amount of patients who received a two-stent treatment for bifurcation disease is small compared to the one who received the provisional approach. Finally, although designed as an all comers study, only 23% of patients undergoing percutaneous interventions were actually enrolled in the study, so selection bias cannot be entirely ruled out.

## Conclusion

The main message is that BP-DES look as safe as DP-DES even in high anatomical risk setting like LM disease. Furthermore, the provisional approach confirms itself as the safer on the long term. Finally, when a two-stent strategy is absolutely needed, the trends toward improved clinical outcomes with respect to MACE and TVR we found with BP-DES might potentially be of value to speculate about the stent choice in selected high-risk subgroups of patients at increased risk of ischemic events.

## Supplementary information

**Additional file 1 Supplementary Methods.** List of leading and participating study centres. Lists of stents used in the study. **Supplementary Table 1**. Baseline and interventional characteristics before the propensity score.

## Data Availability

On request; personal data are protected according to the law. Please refer to the corresponding author at umberto.barbero@unito.it. The datasets used and/or analysed during the current study are available from the corresponding author on reasonable request.

## References

[1] Mintz GS, Lefèvre T, Lassen JF, Testa L, Pan M, Singh J, Stankovic G, Banning AP. Intravascular ultrasound in the evaluation and treatment of left main coronary artery disease: a consensus statement from the European Bifurcation Club. EuroIntervention. 2018;14(4):e467–e474. 10.4244/EIJ-D-18-00194 29688182

[2] D'Ascenzo F, Iannaccone M, Giordana F, Chieffo A, Connor SO, Napp LC, Chandran S, de la Torre Hernández JM, Chen SL, Varbella F, Omedè P, Taha S, Meliga E, Kawamoto H, Montefusco A, Chong M, Garot P, Sin L, Gasparetto V, Abdirashid M, Cerrato E, Biondi-Zoccai G, Gaita F, Escaned J, Hiddick Smith D, Lefèvre T, Colombo A, Sheiban I, Moretti C (2016). Provisional vs. two-stent technique for unprotected left main coronary artery disease after ten years follow up: A propensity matched analysis. Int J Cardiol.

[3] D'Ascenzo F, Chieffo A, Cerrato E, Ugo F, Pavani M, Kawamoto H, di Summa R, Varbella F, Boccuzzi G, Omedè P, Rettegno S, Garbo R, Conrotto F, Montefusco A, Biondi-Zoccai G, D'Amico M, Moretti C, Escaned J, Gaita F, Colombo A (2017). Incidence and Management of Restenosis after Treatment of unprotected left Main disease with second-generation drug-eluting stents (from failure in left Main study with 2nd generation stents-Cardiogroup III study). Am J Cardiol.

[4] Chiastra C, Gallo D, Tasso P, Iannaccone F, Migliavacca F, Wentzel JJ, Morbiducci U (2017). Healthy and diseased coronary bifurcation geometries influence near-wall and intravascular flow: a computational exploration of the hemodynamic risk. Biomech.

[5] Sawaya FJ, Lefèvre T, Chevalier B, Garot P, Hovasse T, Morice MC, Rab T, Louvard Y (2016). Contemporary approach to coronary bifurcation lesion treatment. JACC Cardiovasc Interv..

[6] Yu CW, Yang JH, Song YB, Hahn JY, Choi SH, Choi JH, Lee HJ, Oh JH, Koo BK, Rha SW, Jeong JO, Jeong MH, Yoon JH, Jang Y, Tahk SJ, Kim HS, Gwon HC (2015). Long-term clinical outcomes of final kissing ballooning in coronary bifurcation lesions treated with the 1-stent technique: results from the COBIS II registry (Korean coronary bifurcation stenting registry). JACC Cardiovasc Interv.

[7] Garg S, Wykrzykowska J, Serruys PW, de Vries T, Buszman P, Trznadel S, Linke A, Lenk K, Ischinger T, Klauss V, Eberli F, Corti R, Wijns W, Morice MC, di Mario C, Tyczynski P, van Geuns RJ, Eerdmans P, van Es GA, Meier B, Jüni P, Windecker S (2011). The outcome of bifurcation lesion stenting using a biolimus-eluting stent with a bio-degradable polymer compared to a sirolimus-eluting stent with a durable polymer. EuroIntervention.

[8] Kereiakes DJ, Meredith IT, Windecker S, Lee Jobe R, Mehta SR, Sarembock IJ, Feldman RL, Stein B, Dubois C, Grady T, Saito S, Kimura T, Christen T, Allocco DJ, Dawkins KD. Efficacy and safety of a novel bioabsorbable polymer-coated, everolimus-eluting coronary stent: the EVOLVE II Randomized Trial. Circ Cardiovasc Interv. 2015 Apr;8(4). PubMed PMID: 25855680. 10.1161/CIRCINTERVENTIONS.114.002372. 10.1161/CIRCINTERVENTIONS.114.002372 25855680

[9] Mennuni MG, Stefanini GG, Pagnotta PA, Pllaha E, Araco M, Meelu OA, Turati F, Reimers B, Sardella G, Presbitero P (2017). Clinical outcomes of bioresorbable versus durable polymer-coated everolimus-eluting stents in real-world complex patients. EuroIntervention.

[10] Chen SL, Sheiban I, Xu B (2014). Impact of the complexity of bifurcation lesions treated with drug-eluting stents: the DEFINITION study (definitions and impact of complEx biFurcation lesIons on clinical outcomes after percutaNeous coronary IntervenTIOn using drug-eluting steNts). JACC Cardiovasc Interv.

[11] D'Ascenzo F, Cavallero E, Biondi-Zoccai G, Moretti C, Omedè P, Bollati M, Castagno D, Modena MG, Gaita F, Sheiban I (2012). Use and misuse of multivariable approaches in interventional cardiology studies on drug-eluting stents: a systematic review. J Interv Cardiol.

[12] Kang SH, Park KW, Kang DY (2014). Biodegradable- polymer drug-eluting stents vs. bare metal stents vs. durable-polymer drug-eluting stents: a systematic review and Bayesian approach network meta-analysis. Eur Heart J.

[13] Bangalore S, Hannan EL, Toklu B (2013). Bare metal stents, durable polymer drug eluting stents, and biodegradable polymer drug eluting stents for coronary artery disease: mixed treatment comparison meta-analysis. BMJ.

[14] Navarese EP, Tandjung K, Claessen B (2013). Safety and efficacy outcomes of first and second generation durable polymer drug eluting stents and biodegradable polymer biolimus eluting stents in clinical practice: comprehensive network meta-analysis. BMJ.

[15] Cassese S, Ndrepepa G, Byrne RA, Kufner S, Lahmann AL, Mankerious N, Xhepa E, Laugwitz KL, Schunkert H, Fusaro M, Kastrati A, Joner M. Outcomes of patients treated with ultrathin strut biodegradable-polymer sirolimus-eluting stents versus fluoropolymer-based everolimus-eluting stents. A meta-analysis of randomized trials. EuroIntervention. 2018. 10.4244/EIJ-D-18-00024 [Epub ahead of print]. 10.4244/EIJ-D-18-00024 29537375

[16] Mennuni MG, Stefanini GG, Pagnotta PA, Pllaha E, Araco M, Meelu OA, Turati F, Reimers B, Sardella G, Presbitero P (2017). Clinical outcomes of bioresorbable versus durable polymer-coated everolimus-eluting stents in real-world complex patients. EuroIntervention.

[17] El-Hayek G, Bangalore S, Casso Dominguez A, Devireddy C, Jaber W, Kumar G, Mavromatis K, Tamis-Holland J, Samady H (2017). Meta-analysis of randomized clinical trials comparing biodegradable polymer drug-eluting stent to second-generation durable polymer drug-eluting stents. JACC Cardiovasc Interv.

[18] D'Ascenzo F, Iannaccone M, Saint-Hilary G, Bertaina M, Schulz-Schüpke S, Wahn Lee C, Chieffo A, Helft G, Gili S, Barbero U, Biondi Zoccai G, Moretti C, Ugo F, D'Amico M, Garbo R, Stone G, Rettegno S, Omedè P, Conrotto F, Templin C, Colombo A, Park SJ, Kastrati A, Hildick-Smith D, Gasparini M, Gaita F (2017). Impact of design of coronary stents and length of dual antiplatelet therapies on ischaemic and bleeding events: a network meta-analysis of 64 randomized controlled trials and 102 735 patients. Eur Heart J.

[19] Kolandaivelu K, Swaminathan R, Gibson WJ (2011). Stent thrombogenicity early in high-risk interventional settings is driven by stent design and deployment and protected by polymer-drug coatings. Circulation.

[20] Vlachojannis GJ, Smits PC, Hofma SH, Togni M, Vázquez N, Valdés M, Voudris V, Slagboom T, Goy JJ, den Heijer P, van der Ent M (2017). Biodegradable polymer Biolimus-eluting stents versus durable polymer Everolimus-eluting stents in patients with coronary artery disease: final 5-year report from the COMPARE II trial (Abluminal biodegradable polymer Biolimus-eluting stent versus durable polymer everolimus-eluting stent). JACC Cardiovasc Interv.

[21] Cassese S, Lahmann AL, Joner M (2018). Ultrathin strut biodegradable-polymer sirolimus-eluting stents: being wary or going with the flow?. J Thorac Dis.

[22] Kandzari DE, Mauri L, Koolen JJ, Massaro JM, Doros G, Garcia-Garcia HM, Bennett J, Roguin A, Gharib EG, Cutlip DE, Waksman R (2017). BIOFLOW V investigators. Ultrathin, bioresorbable polymer sirolimus-eluting stents versus thin, durable polymer everolimus-eluting stents in patients undergoing coronary revascularisation (BIOFLOW V): a randomised trial. Lancet.

[23] Chen SL, Xu B, Han YL, Sheiban I, Zhang JJ, Ye F, Kwan TW, Paiboon C, Zhou YJ, Lv SZ, Dangas GD, Xu YW, Wen SY, Hong L, Zhang RY, Wang HC, Jiang TM, Wang Y, Sansoto T, Chen F, Yuan ZY, Li WM, Leon MB (2015). Clinical outcome after DK crush versus Culotte stenting of distal left Main bifurcation lesions: the 3-year follow-up results of the DKCRUSH-III study. JACC Cardiovasc Interv.

[24] Kok MM, Zocca P, Buiten RA, Danse PW, Schotborgh CE, Scholte M, Hartmann M, Stoel MG, van Houwelingen G, Linssen GCM, Doggen CJM, von Birgelen C. Two-year clinical outcome of all-comers treated with three highly dissimilar contemporary coronary drug-eluting stents in the randomised BIO-RESORT trial. EuroIntervention. 2018. 10.4244/EIJ-D-18-00336 [Epub ahead of print]. 10.4244/EIJ-D-18-00336 29790480

[25] Smits PC, Vlachojannis GJ, McFadden EP (2015). Final 5-year follow-up of a randomized controlled trial of everolimus- and paclitaxel-eluting stents for coronary revascularization in daily practice: the COMPARE trial (a trial of Everolimus-eluting stents and paclitaxel stents for coronary revascularization in daily practice). J Am Coll Cardiol Intv.

[26] Barbero U, Kanji R, Cerrato E, Di Summa R, Conrotto F, Kawamoto H, Biondi-Zoccai G, Gili S, Ugo F, Iannaccone M, Gagliardi M, De Benedictis M, Doronzo B, Varbella F, D'Amico M, Moretti C, Colombo A, Escaned J, D'Ascenzo F (2018). Unprotected left Main coronary artery disease: outcomes of treatment with second-generation drug-eluting stents - insight from the FAILS-2 study. J Invasive Cardiol.

[27] Serruys PW, Farooq V, Kalesan B (2013). Improved safety and reduction in stent thrombosis associated with biodegradable polymer-based biolimus-eluting stents versus durable polymerbased sirolimus-eluting stents in patients with coronary artery disease: final 5-year report of the LEADERS (Limus eluted from a durable versus ERodable stent coating) randomized, noninferiority trial. J Am Coll Cardiol Intv.

[28] D'Ascenzo F, Barbero U, Cerrato E, Lipinski MJ, Omedè P, Montefusco A, Taha S, Naganuma T, Reith S, Voros S, Latib A, Gonzalo N, Quadri G, Colombo A, Biondi-Zoccai G, Escaned J, Moretti C, Gaita F (2015). Accuracy of intravascular ultrasound and optical coherence tomography in identifying functionally significant coronary stenosis according to vessel diameter: a meta-analysis of 2,581 patients and 2,807 lesions. Am Heart J.

[29] Valgimigli M, Bueno H, Byrne RA, Collet JP, Costa F, Jeppsson A, Juni P, Kastrati A, Kolh P, Mauri L, Montalescot G, Neumann FJ, Petricevic M, Roffi M, Steg PG, Windecker S, Zamorano JL, Levine GN (2018). 2017 ESC focused update on dual antiplatelet therapy in coronary artery disease developed in collaboration with EACTS: The Task Force for dual antiplatelet therapy in coronary artery disease of the European Society of Cardiology (ESC) and of the European Association for Cardio-Thoracic Surgery (EACTS). Eur Heart J.

[30] D'Ascenzo F, Colombo F, Barbero U, Moretti C, Omedè P, Reed MJ, Tarantini G, Frati G, Di Nicolantonio JJ, Biondi Zoccai G, Gaita F (2014). Discontinuation of dual antiplatelet therapy over 12 months after acute coronary syndromes increases risk for adverse events in patients treated with percutaneous coronary intervention: systematic review and meta-analysis. J Interv Cardiol.

[31] Gili S, Barbero U, Errigo D, De Luca G, Biondi-Zoccai G, Leone AM, Iannaccone M, Montefusco A, Omedé P, Moretti C, D'Amico M, Gaita F, D'Ascenzo F (2018). Intracoronary versus intravenous adenosine to assess fractional flow reserve: a systematic review and meta-analysis. J Cardiovasc Med (Hagerstown).

