# Supplementary material for: Comparison of bioresorbable vs durable polymer drug-eluting stents in unprotected left main (from the RAIN-CARDIOGROUP VII Study)
Source: BMC Cardiovasc Disord. 2020 May 15;20:225. doi: 10.1186/s12872-020-01420-5 (PMC7227223; doi:10.1186/s12872-020-01420-5)
Supplement: Supplementary file 1 — Additional file 1 Supplementary Methods. List of leading and participating study centres. Lists of stents used in the study. Supplementary Table 1. Baseline and interventional characteristics before the propensity score. [file 12872_2020_1420_MOESM1_ESM.doc]

*Appendix, web only*

**Comparison of Bioresorbable vs Durable polymer drug-eluting stents on ULM**

A propensity score with matching from the RAIN-CARDIOGROUP VII study (veRy thin stents for patients with left mAIn or bifurcatioN in real life)

**Table of Contents**

Supplementary Methods …………………………………………………………, 3

Definitions………………….………………………………………………………… 4

Supplementary Table 1 ………………………………………………………… .5

References……………….………………………………………………………… 6

**Supplementary Methods.**

**Leading Study Center**

Dipartimento di Scienze Mediche, Divisione di Cardiologia, Città della Salute e della Scienza, Turin, Italy

**Participating Study Centers**

Division of Cardiology, Department of Internal Medicine, Città della Salute e della Scienza, University of Turin, Italy

Coronary Care Unit and Catheterization laboratory , A.O.U. Maggiore della Carità , Novara , Italy

Structural Interventional Cardiology, Careggi University Hospital, Florence, Italy

Division of Cardiology, Cardio-Thoracic-Vascular Department, Azienda Ospedaliero Universitaria "Policlinico-Vittorio Emanuele," Catania, Italy

Department of Cardiovascular Medicine, Nippon Medical School, 1-1-5, Sendagi, Bunkyo-ku, Tokyo, Japan

Department of Cardiovascular Sciences, IRCCS Centro Cardiologico Monzino, Milan, Italy; University of Milan, Milan, Italy

Interventional Cardiology, ASST Fatebenefratelli-Sacco, Milano, Italy

Dipartimento di Cardiologia, Ospedale San Giovanni Bosco, Italy

University Clinical Hospital, Warsaw, Poland

Department of Cardiology, Hospital Clinico San Carlos, Madrid, Spain

San Raffaele Scientific Institute, Milan, Italy

Pederzoli Hospital, Peschiera del Garda, Italy

Department of Cardiology, Medical University of Silesia, Katowice, Poland

Department of Cardiology, Infermi Hospital, Rivoli, Italy

Department of Cardiology, San Luigi Gonzaga Hospital, Orbassano, Turin, Italy

Division of Cardiology, S. Giovanni Evangelista Hospital, Tivoli, Rome, Italy

Pierre and Marie Curie University, Paris, France

Division of Cardioloy, Universityspirtal of Zurich

**Definitions.**

- **Critical stenosis of Left Main (LM)** was defined, according to guidelines, as visual stenosis of more than 50% at coronary angiography, or critical area at intravascular ultrasound evaluation or positive values of fractional flow reserve, according to evaluation of Physician at Single Institution (1)
- **Complex bifurcations were defined according to the following criteria (major + any 2 minor) (2)**

1. Major: Distal LM bifurcation SB (Side Branch)-DS (Diameter stenosis)>70% and SB lesion length>=10 mm.
2. Minor:

- Moderate to severe calcification
- Multiple lesions
- Bifurcation angle<45°
- Main vessel RVD>2.5 mm
- Thrombus containing lesions
- MV lesione lenght >=25 mm

**Stents:**

- Platinum-chromium coated with a durable polymer loading everolimus with strut thickness of 81 µm for diameters 2.25-3.5 mm (Promus Element, Boston Scientific);
- Cobalt-chromium coated with a durable polymer loading everolimus with a strut thickness of 80 µm (Xience Alpine, Abbot);
- Cobalt-chromium coated with a biodegradable polymer loading sirolimus with strut thickness of 80 µm; (Ultimaster, Terumo Corporation);
- Platinum-chromium coated with a biodegradable polymer loading everolimus with strut thickness of 74 µm for diameters 2.25-2.75 mm, 79 µm for diameters 3.00-3.50 mm, and 81 µm for the diameter of 4.0 mm; (Synergy, Boston Scientific);
- Platinum-chromium coated with a durable polymer loading zotarolimus with a strut thickness of 74 µm for diameters ≤2.5 mm, 79 µm for diameters 3.0-3.50 mm, and 81 µm for diameters from 4.0 mm (Resolute Onyx, Medtronic).

**Additional file 1 TABLE A** - Baseline Characteristics before PSWM

|  | **DP-DES**  **(2120 pt)** | **BP-DES**  **(881 pt)** | **P** |
| --- | --- | --- | --- |
| *Age* | 70.82±9 | 70.76±10.6 | 0.974 |
| *Female (%)* | 23.3 | 24.6 | 0.45 |
| *Hypertension (%)* | 74.9 | 74.1 | 0.67 |
| *Hyperlipidemia (%)* | 58.1 | 64.6 | <0.01 |
| *Diabete mellitus non ID (%)* | 23.2 | 28.8 | <0.01 |
| *Diabete mellitus ID (%)* | 6.8 | 9.3 | 0.04 |
| *Previous smoker (%)* | 29.1 | 30 | 0.09 |
| *Current Smoker* | 5 (11.4) | 106 (14.5) | 0.753 |
| *Renal Disease (gfr <60 ml/min/m2) (%)* | 73.2 | 70.7 | 0.24 |
| *Previous PCI (%)* | 32.1 | 31.9 | 0.89 |
| *Previous CABG (%)* | 5.3 | 4.1 | 0.19 |
| *Previous MI (%)* | 26.6 | 35.3 | <0.01 |
| *ASA + Clopidogrel (%)* | 65.9 | 68.7 | 0.11 |
| *ASA + Ticagrelor (%)* | 24.5 | 24.3 | 0.23 |
| *ASA + Prasugrel (%)* | 7.3 | 6.3 | 0.35 |
| *Length of DAPT (months)* | 11.17 | 11.81 | 0.13 |
| *Indication for PCI: (%)*   - *STEMI* - *NSTEMI* - *UA* - *Stable angina* - *Planned angiographic follow up* | *16*  22.5  14.5  26.5  12.9 | 19.1  27.7  13.6  23.5  11.5 | <0.01 |

**Additional file 1 Table B** - **Interventional Characteristics before PSWM**

|  | **DP-DES**  **(2120 pt)** | **BP-DES**  **(881 pt)** | **P** |
| --- | --- | --- | --- |
| *Radial access (%)* | 68.8 | 68.4 | 0.79 |
| *Overall LM (%)* | 30 | 26.9 | 0.45 |
| *Site of lesion:*   - *Ostial LM* - *Mid LM* - *Distal LM* | 15.5  19  65.6 | 15.2  22.8  62 | 0.45 |
| *Type C lesion (%)* | 38.4 | 39.5 | 0.6 |
| *Severe calcification (%)* | 12.9 | 13.9 | 0.49 |
| *Diffuse disease (%)* | 29.9 | 55.1 | <0.01 |
| *All bifurcation site*   - *Distal LM* - *LAD/Dg* - *LCx/OM* - *RCA/Pl* | 28.3  46.3  17.8  7.7 | 27.6  47.8  15.7  8.9 | 0.14 |
| *True bifurcation ( medina 1,1,1 or 0,1,1)* | 17.5 | 28.1 | <0.01 |
| *Provisional strategy (%)* | 82.4 | 76.9 | <0.01 |
| *2 stents technique strategy (%)*   - *Culotte* - *Mini crush* - *Crush* - *DK-crush* - *T stent* - *TAP stent* | 1.7  3.1  0.5  0.3  4.4  2.9 | 1.6  5  0.8  0.6  2.8  3.7 | 0.08 |
| *Use of imaging:*   - *IVUS* - *OCT* | 31.1  0.7 | 35.8  1.8 | <0.01 |

**REFERENCES.**

1. StephanWindecker, Kolh P, Alfonso F, Collet JP, Cremer J, Falk V, Filippatos G, Hamm C, Head SJ, Jüni P, Kappetein AP, Kastrati A, Knuuti J, Landmesser U, Laufer G, Neumann FJ, Richter DJ, Schauerte P, Uva MS, Stefanini GG, Taggart DP, Torracca L, Valgimigli M, Wijns W, Witkowski A. 2014 ESC/EACTS guidelines on myocardial revascularization. Rev Esp Cardiol (Engl Ed). 2015 Feb;68(2):144.
2. Chen SL, Sheiban I, Xu B, et al. Impact of the complexity of bifurcation lesions treated with drug-eluting stents: the DEFINITION study (Definitions and Impact of Complex Bifurcation Lesions on Clinical Outcomes After Percutaneous Coronary Intervention Using Drug-Eluting Stents). J Am Coll Cardiol Intv 2014;7:1266–76
